# Retraction: APPL1-Mediating Leptin Signaling Contributes to Proliferation and Migration of Cancer Cells

**DOI:** 10.1371/journal.pone.0283346

**Published:** 2023-03-15

**Authors:** The PLOS ONE Editors

Following the publication of this article [1], concerns were raised regarding results presented in Figs 1, 4, 5, 6, and 7. Specifically,

The following panels appear similar:

- The Fig. 4A HepG2 Tubulin and MCF Tubulin panels.
- The Fig. 7A HepG2 Tubulin and MCF Tubulin panels.
- Lanes 1–4 of the Fig. 1D Tubulin panel of this study [1] and the Fig. 1D Tubulin panel of [2].
- The Fig. 4A MCF p-AKT panel of this study [1], lanes 3–6 of the Fig. 4A p-STAT3 panel of [3], and the Fig. 2B p-AS160 panel of [4].
- The Fig. 4A MCF7 ERK panel of this study [1] and lanes 1–4 of the Fig. 6D ERK1/2 panel of [5, note of concern published in 6].
- Lanes 1–3 of the Fig. 5A MCF7 p-JAK2 of this study [1] and the Fig. 5D OPN panel of [7] when flipped horizontally.
- Lanes 1–2 of the Fig. 6A MCF7 Input STAT3 panel of this study [1] and lanes 2–3 of the Fig. S2 STAT3 panel of [3].
- The Fig. 6B MCF7 Lysate p-STAT3 panel of this study [1] and lanes 2–5 of the Fig. 4A GRP78 panel of [3] when flipped horizontally.
- Lanes 2–4 of the Fig. 6B HepG2 Lysate p-STAT3 panel of this study [1] and the Fig. 8C p62 panel of [8].
- The Fig. 6B MCF7 STAT3 panel of this study [1] and the Fig. 7E Tubulin panel of [4].
- The Fig. 6B HepG2 Lysate APPL1 panel of this study [1] and the Fig. 3G PDK1 panel of [9].
- Lanes 2–5 of the Fig. 7A HepG2 p-STAT3 panel of this study [1] and the Fig. 5C Akt panel of [8].
- Lanes 1–3 of the Fig. 7A MCF7 p-AKT panel of this study [1] and the Fig. S2 p-STAT3 panel of [3].
- Lanes 1–4 of the Fig. 7A MCF7 AKT panel of this study [1] and the Fig. 3D Tubulin panel of [8].

The corresponding author commented that some western blot images were misused in this article [1] and requested the retraction of the article.

In light of the concerns affecting multiple figure panels that question the integrity of these data, the *PLOS ONE* Editors retract this article.

CW agreed with the retraction. YD, YC, BW, LW, YZ, DZ, XC, and ML either did not respond directly or could not be reached.
